# Potential Antioxidant Activity of Calcium and Selected Oxidative Stress Markers in Lead- and Cadmium-Exposed Workers

**DOI:** 10.1155/2020/8035631

**Published:** 2020-10-05

**Authors:** Zbigniew Szlacheta, Marta Wąsik, Anna Machoń-Grecka, Aleksandra Kasperczyk, Michał Dobrakowski, Francesco Bellanti, Patryk Szlacheta, Sławomir Kasperczyk

**Affiliations:** ^1^Upper Silesian Rehabilitation Centre “Repty”, Tarnowskie Góry, Poland; ^2^Department of Clinical Biochemistry and Laboratory Diagnostics, Institute of Medicine, University of Opole, Oleska 48, 45-052 Opole, Poland; ^3^Department of Biochemistry, Faculty of Medical Sciences in Zabrze, Medical University of Silesia in Katowice, Poland; ^4^Department of Medical and Surgical Sciences, University of Foggia, Viale Pinto 1, 71122 Foggia, Italy; ^5^Department of Toxicology and Health Protection, Faculty of Health Science in Bytom, Medical University of Silesia in Katowice, Bytom, Poland

## Abstract

Occupational lead (Pb) and cadmium (Cd) exposure occurs during processing and casting of nonferrous metals such as zinc. In contrast to Pb and Cd, Ca is essential for living organisms due to its important role in a multitude of functions, from cell signaling to bone growth. Pb and Cd exposure affects calcium metabolism in various ways. The aim of this study was to investigate the blood levels of Pb, Cd, and Ca and the levels of selected oxidative stress biomarkers in workers exposed to Pb and Cd. Population groups included 264 male employees in a lead-zinc smelter. The study population was divided into two subgroups based on the median of Ca serum level (2.42 mmol/l): the low-Ca-level group (L-Ca group) and the high-Ca-level group (H-Ca group). Ca level was significantly higher in the H-Ca group than in the L-Ca group due to the study design (by 26%). The level of zinc protoporphyrin (ZPP) was significantly higher in the L-Ca group than in the H-Ca group by 13%, while the blood lead levels (PbB) were similar in the examined groups. The level of cadmium (CdB) was significantly higher in the L-Ca group than in the H-Ca group by 33%. From oxidative stress markers in serum, only the levels of malondialdehyde (MDA) and ceruloplasmin (CER) were significantly higher in the L-Ca group than in the H-Ca group, by 12% and 4%, respectively. The correlation analysis showed negative correlations between Ca level and the levels of PbB, ZPP, CdB, and MDA. The presented results indicate that Ca level modulates the serum concentration of Cd and has an impact on Pb-induced impairment of heme synthesis. The higher Ca levels may lead to a decrease in the concentration of lipid peroxidation products. Moreover, serum calcium level seems to be able to modify the level of acute-phase proteins. Obtained results suggest that higher Ca level may be useful in reducing Cd level in occupationally exposed workers.

## 1. Introduction

Lead (Pb) and cadmium (Cd), potent toxic heavy metals, are widespread environmental contaminants. The common sources are diverse in nature and include natural and anthropogenic processes. The World Health Organization (WHO) has included these two toxic metals in the top 10 chemicals of major public health concern [1]. Occupational Cd and Pb exposure occurs during processing and casting of nonferrous metals such as zinc [2]. Pb and Cd are common contaminants in zinc-bearing ores, and overexposure to these metals may occur. Pb exposure and lead toxicity are a particularly important problem in the construction industry. The Occupational Safety and Health Administration (OASA) estimates that nearly 1 million U.S. construction workers are exposed to lead on the job. Estimates indicate that more than 0.5 million workers are exposed to Cd in U.S. workplaces [3]. In 2004-2005, in Poland, 3297 employees were working in conditions of exposure to Pb at concentrations higher than the maximum allowable concentration (MAC) in the air that is 0.050 mg/m^3^ [4]. In lead-zinc smelters, the major toxic exposure in Pb is lead dust, but there is also exposure to zinc, cadmium, arsenic, and thallium dust and vapors. Cd and Pb admissible concentrations in biological material (DSB) are 5 *μ*g/l and 50 *μ*g/dl, respectively [5].

Lead poisoning can cause anemia, hypertension, risk for stroke and cardiovascular disease, and central and peripheral neurotoxicity [6]. Some indicators of a biological effect of Pb on heme synthesis are the activity of the enzyme 5-aminolevulinic acid dehydratase in blood, the concentrations of 5-aminolevulinic acid and coproporphyrin in urine, and the concentration of porphyrins in blood [7]. It has been shown that the concentration of porphyrins, specifically zinc protoporphyrin (ZPP), in blood offers the best predictor of the level of lead in blood among workers [8].

It can be observed that Cd^2+^, which belongs to group 12 in the periodic table of elements, is classified as soft metal ions, preferring the coordination by ligands characterized by soft groups such as R_2_S, RSH, and RS [9]. Lead cation, which belongs to group 14 in the periodic table, is classified as an intermediate metal ion, indicating that above all it will be coordinated by amino groups, even if the interaction with hard oxygen groups and soft thiol groups is observed in a number of complexes [10]. Furthermore, Pb^2+^ and Cd^2+^ also have a similar ionic radius: 1.19 Å and 1.09 Å as calcium ion (1.00 Å). Both can displace Ca^2+^ in proteins (with Ca^2+^-binding sites) and alter the homeostasis of this cation [11, 12].

The cellular uptake mechanisms of Pb and Cd are still not clear. It is hypothesized that Cd^2+^ uptake involves competition with Ca^2+^, Fe^2+^, and Zn^2+^ and makes use of their transport systems [13]. Cd may enter the apical membrane of intestinal epithelial cells through the divalent metal transporter 1 (DMT1) and through zinc transporters such as ZIP1, ZIP2, ZIP8, and ZIP14 [14]. Pb^2+^ ions can also mimic Fe^2+^ and Ca^2+^ to gain access to the intracellular compartment using their transporters, but this uptake is dependent of the cell type and pH changes [15].

Ca^2+^ and both Pb^2+^ and Cd^2+^ are linked with oxidative stress but on different pathways. Oxidative stress is described as an imbalance between the production of free radicals and the biological system's ability to readily detoxify the reactive intermediates. It has been reported that lead-induced oxidative stress occurs by two different pathways. On the one hand, Pb stimulates the generation of Reactive Oxygen Spaces (ROS), while on the other hand, it depletes both the enzymatic and nonenzymatic antioxidant reserves [16, 17]. Pb and Cd induce oxidative stress by a displacement of redox-active metals, e.g., by the depletion of redox scavengers or by the inhibition of antioxidant enzymes [13]. Some studies suggested that higher Ca levels stimulate antioxidants and mitigate the impacts of metals [18]. On the other hand, mitochondrial Ca^2+^ may promote ROS formation both directly, by stimulating ROS-generating enzymes, and indirectly, as in the case of nitric oxide synthase (NOS) activation that, by forming NO, blocks complex IV, leading to excessive ROS formation [19].

Ca is the most abundant stored nutrient in the human body. More than 99% (1.2-1.4 kg) is stored in the bones and teeth. Less than 1% is found in extracellular serum calcium [20]. In contrast to Pb and Cd, Ca is essential for living organisms due to its important role in a multitude of functions, from cell signaling to bone growth [21]. Serum calcium does not fluctuate with changes in dietary intake. The smallest drop in serum Ca below the normal level will trigger an immediate response. Cd and Pb mimic Ca at their specific sites and bind to calmodulin, protein kinase C, and synaptic proteins [22]. Pb and Cd exposure affects calcium metabolism in various ways. Furthermore, some studies suggested that calcium levels may have an impact on the blood lead levels [23–25].

There is limited information in the available literature on the relationships between blood lead, cadmium, and calcium levels and oxidative stress in occupationally exposed workers.

In light of this, the present study was undertaken to examine the blood levels of Pb, Cd, and Ca and the levels of selected oxidative stress biomarkers in workers exposed to Pb and Cd.

## 2. Materials and Methods

### 2.1. Study Population

Study protocol was approved by the Bioethics Committee of the Medical University of Silesia (Permission No. KNW/022/KB1/108/14).

Population groups included 264 male employees in the lead-zinc smelter in Miasteczko Śląskie, Poland. The smelter produces zinc and lead by pyrometallurgical extraction using the Imperial Smelting Process (ISP) which is based on the reduction of roasted Zn-Pb concentrate with coke at 1000°C in a shaft furnace. All of the Pb- and Cd-exposed subjects were employed in the same lead-zinc works as smelters, fitters, and production masters. Questionnaire data on age, weight, height, duration of employment under lead and cadmium exposure, medical history (chronic diseases such as diabetes, coronary artery disease, and arterial hypertension), and smoking were obtained. The levels of lead (PbB) and zinc protoporphyrin (ZPP) in the blood served as biomarkers of lead exposure. Cadmium exposure was estimated based on blood levels of cadmium. Inclusion criteria for the study were occupational exposure to Pb and PbB exceeding 20 *μ*g/dl. Exclusion criteria included a history of any chronic diseases (without diabetes, coronary artery disease, and arterial hypertension) and abnormal physical examination findings, especially symptoms and signs of any infectious diseases.

The study population was divided into two subgroups based on the median of the serum Ca level (2.42 mmol/l): the low-calcium-level group (L-Ca group, *n* = 132) and the high-Ca-level group (H-Ca group, *n* = 132). Serum Ca level ranged from 2.42 to 3.75 mmol/l in the H-Ca group and from 1.57 to 2.41 mmol/l in the L-Ca group.

This work was supported by the Medical University of Silesia (Grant No. KNW-1-045/K/8/K).

### 2.2. Laboratory Procedures

Blood samples were collected from a peripheral vein using tubes (Vacuette; Greiner Bio-One, Frickenhausen, Germany) coated with K_3_EDTA to obtain whole blood or plain tubes to obtain serum. Serum was collected using centrifugation (3,000 rpm for 10 min at 4°C), aliquoted, and stored at -80°C until analysis.

### 2.3. Concentrations of Lead (Pb), Cadmium (Cd), Zinc Protoporphyrin (ZPP), and Calcium (Ca)

Assessments of the Pb and Cd levels were performed using graphite furnace atomic absorption spectrometry in an iCE 3400 system. The results were expressed as *μ*g/dl for the Pb level and *μ*g/l for the Cd level. The blood concentration of zinc protoporphyrin (ZPP) was measured directly using an Aviv Biomedical hematofluorometer model 206, using an excitation wavelength of 415 nm and an emission wavelength of 596 nm. The instrument measures the ratio of ZPP fluorescence to the sample (hemoglobin) absorption, displayed as *μ*g ZPP per g of hemoglobin (*μ*g/g Hb). The concentration of hemoglobin in 10% hemolysate was determined by the cyanmethemoglobin method using Drabkin's reagent. Assessments of the Ca levels were performed using graphite furnace atomic absorption spectrometry in an iCE 3400 (Thermo Fisher Scientific, Waltham, MA, USA). The results were expressed as mmol/l.

### 2.4. Blood Morphology

The Sysmex K-4500 analyzer (GMI, MN, USA) was used to determine the parameters of blood morphology: white blood cell (WBC) count, red blood cell (RBC) count, hemoglobin (HGB) level, hematocrit (HTC), and platelet (PLT) count. We followed the methods of Wyparło-Wszelaki et al. [26].

### 2.5. Markers of Antioxidant Defense and Oxidative Stress

Total antioxidant capacity (TAC) was measured in serum according to [27]. This method involves the synthesis of free radicals, which are reduced by antioxidants contained in the samples. This process results in a color change of the ABTS+ ion (2,2′-azino-bis (3-ethyl-benzothiazoline-6-sulfonic acid) diammonium salt). Data were expressed as mmol/l. Total oxidant status (TOS) was measured in serum according to Erel [28]. This method is based on the oxidation of Fe^2+^ to Fe^3+^ in the oxidizing agents in an acid medium, resulting in a color change of xylenol orange measured as a change in absorbance at 560 nm. Data were expressed as *μ*mol/l. Oxidative stress index (OSI) was calculated as the percentage ratio of TOS to TAC. Lipid hydroperoxides (LPH) in serum were measured according to [29] using xylene orange. The results were shown in *μ*mol/l. The level of malondialdehyde (MDA), a product of lipid peroxidation, was measured fluorometrically as a 2-thiobarbituric acid-reactive substance (TBARS) in serum and erythrocytes according to Ohkawa et al. [30] with the same modifications. Tetraethoxypropane was used as the standard. Concentrations were given in *μ*mol/l serum and in nmol/gHb erythrocytes. The concentration of lipofuscin (LPS) was measured according to the method of Tsuchida et al. [31]. The results were expressed as relative units (RU) per gram of hemoglobin in erythrocytes and in RU/l in serum (the fluorescence of a 0.1 mg/ml solution of quinidine sulfate in sulfuric acid is equal to 100 RU). Sulfhydryl group (SH) concentration was determined as described by Koster et al. [32] using 5,5′-dithiobis (2-nitrobenzoic acid) (DTNB), which is reduced by compounds containing sulfhydryl groups to give a yellow-colored 5-thio-2-nitrobenzoic anionic derivative. The results were shown in *μ*mol/l. Ceruloplasmin (CER) concentration in serum was determined as described by Richterich [33] using the reaction with p-phenyldiamine. The results were shown in mg/dl. The method of Wyparło-Wszelaki et al. was selected for this study [26].

### 2.6. Antioxidant Enzymes

The activities of superoxide dismutase (SOD) were measured in serum and erythrocytes according to Oyanagiu [34]. The superoxide anion produced by xanthine oxidase (XO) reacts with hydroxylamine to form a nitroso ion which, combined with naphthylenediamine and sulfoanilinic acid, produces a color combination. The enzymatic activity of SOD was expressed in nitric units. The activity of SOD is equal to 1 nitric unit (NU) when it inhibits nitric ion production by 50%. The activities of SOD were normalized to milligrams of Hb (NU/mg Hb). Catalase (CAT) activity in erythrocytes was measured by the kinetic method of Johansson and Håkan Borg [35], which is based on the reaction of CAT with H_2_O_2_ solution at 240 nm. The activity of CAT was expressed as kIU/g Hb. The activity of glutathione reductase (GR) in erythrocytes was measured according to Richterich [33]. GR activity was assayed by the kinetic method, and the decrease of the concentration of nicotin adenosine dinucleotide phosphatase (NADPH+H^+^) after reduction of glutathione disulfide (GSSG) back to glutathione (GSH) was measured. The activity was expressed as *μ*moles of NADPH utilized per minute per g hemoglobin in erythrocytes (IU/g Hb). The activity of glutathione S-transferase (GST) in erythrocytes was measured according to the kinetic method of Habig and Jakoby [36], and the activity of GST was expressed as *μ*moles of thioether produced per minute per g hemoglobin in erythrocytes (mIU/g Hb). Glutathione peroxidase (GPX) activity in erythrocytes was measured by the kinetic method of Paglia and Valentine [37]. Glutathione peroxidase activity in erythrocytes was assayed by the kinetic method [26]. Briefly, reduced GSH is oxidated by H_2_O_2_ to GSSG, and then, glutathione reductase is recovered back to GSH using NADPH+H^+^. The decrease in absorbance is measured at 340 nm. The activity of GPX was expressed as micromoles of NADPH oxidized per minute per g hemoglobin in erythrocytes (IU/g Hb). The method of Wyparło-Wszelaki et al. was selected for this study [26].

### 2.7. Statistical Analysis

All statistical analyses were performed using the Statistica 10.0 PL software program (StatSoft Polska, Warsaw, Poland). All data were reported as means ± SD. A Shapiro-Wilk's test was used to verify normality, and a Levene's test was used to verify homogeneity of variances. Statistical comparisons were made using a *t*-test, a *t*-test with separate variance estimates, a Mann-Whitney *U*-test, or a Chi-squared test. Dependent variables were analyzed using Student's *t*-test and Wilcoxon's test. The Spearman nonparametric correlation was calculated. A *p* value < 0.05 was considered statistically significant.

## 3. Results

### 3.1. Epidemiologic Data and Blood Lead, Cadmium, and Calcium Levels

**Figure 1:**
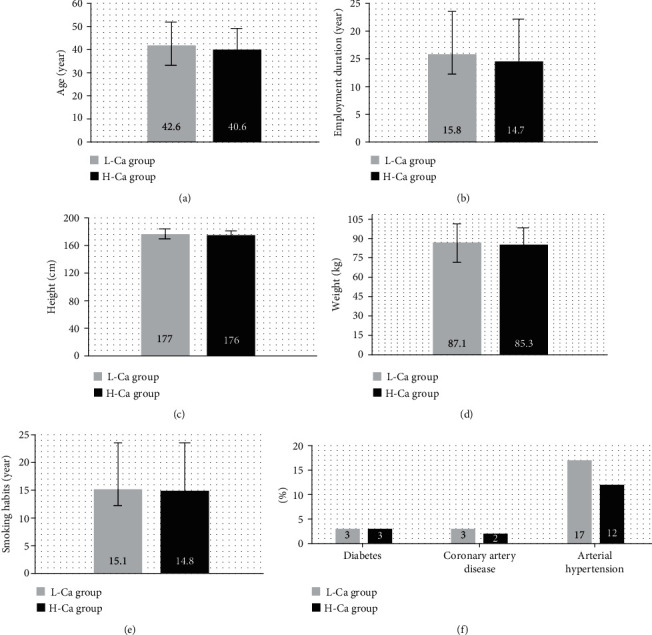
Epidemiologic data (a–e) and percentages of chronic diseases (f) in the low-calcium-level group (L-Ca group) and the high-calcium-level group (H-Ca group). Data are expressed as mean ± standard error or percentages. *p* > 0.05.

**Figure 2:**
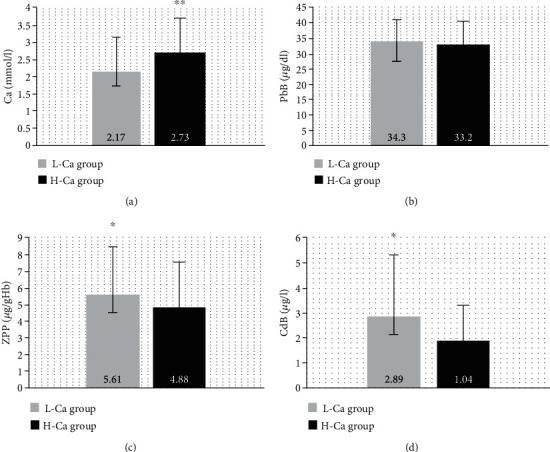
Calcium concentration (a), lead concentration (b), zinc protoporphyrin concentration (c), and cadmium concentration (d) in the low-calcium-level group (L-Ca group) and the high-calcium-level group (H-Ca group). Data are expressed as mean ± standard error. ^∗^*p* < 0.05 and ^∗∗^*p* < 0.001. PbB: blood lead level. ZPP: zinc protoporphyrin. CdB: blood cadmium level.

There were no significant differences in age, employment duration, height, weight, smoking habits, and chronic diseases between the low-calcium-level group (L-Ca group) and the high-calcium-level group (H-Ca group; Figure 1). Calcium level was significantly higher in the H-Ca group than in the L-Ca group due to the study design (by 26%). The level of ZPP was significantly higher in the L-Ca group than in the H-Ca group by 13%, while the PbB were similar in the examined groups. The level of cadmium was significantly higher in the L-Ca group than in the H-Ca group by 33% (Figure 2).

### 3.2. The Study Parameter Concentration Results

**Figure 3:**
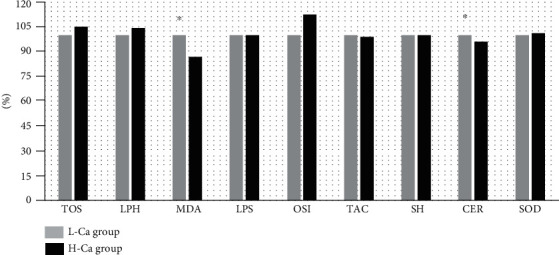
The concentrations of total antioxidant status (TOS), lipid hydroperoxides (LPH), malondialdehyde (MDA), lipofuscin (LPS), oxidative stress index (OSI), total antioxidant capacity (TAC), sulfhydryl groups (SH), ceruloplasmin (CER), and superoxide dismutase (SOD) in serum. Concentrations are expressed as percentage of the mean baseline values in the low-calcium-level group (L-Ca group). ^∗^*p* < 0.05—comparison between the low- and high-calcium-level groups.

The measured parameters of blood morphology were not different between the L-Ca group and the H-Ca group (Table 1). From oxidative stress markers in serum, only the levels of MDA and CER were significantly higher in the L-Ca group than in the H-Ca group, by 12% and 4%, respectively (Figure 3). There was no significant difference in erythrocyte oxidative stress markers between the examined groups (Table 2).

The correlation analysis showed negative correlations between calcium level and the levels of PbB (*R* = −0.13, *p* = 0.034), ZPP (*R* = −0.16, *p* = 0.008), CdB (*R* = −0.24, *p* = 0.001), and MDA (*R* = −0.14, *p* = 0.025; Table 3).

## 4. Discussion

Calcium is the most abundant mineral in the human body and is vital for life. Muscle function, nerve activity, and bone mineralization depend on a precise balance of extracellular and intracellular calcium levels. Under normal circumstances, serum calcium is maintained at a concentration of 8.5–10.5 mg/dl. Even small fluctuations in calcium levels result in significant health consequences [38]. Several studies indicate cadmium-induced calcium homeostasis disruption. In these studies, cadmium causes glomerular and tubular renal dysfunction [13], increases calciuria [14], and is also associated with osteoporosis [39]. Occupational and environmental exposure to Cd has long been associated with increased urinary calcium excretion, which is caused by Cd-induced impairment of tubular reabsorption [40, 41]. Numerous studies are aimed at investigating the hazard of occupational Cd exposure on bone health. In a cross-sectional study of close to 1700 environmentally exposed persons in Belgium, significant associations were found between cadmium dose and an increased urinary excretion of calcium [42]. In our study, we demonstrated, higher serum Cd level in the L-Ca group compared to the H-Ca group. Moreover, we reported a negative correlation between the levels of calcium and cadmium in the examined groups. This data confirms the observations that low Ca levels may enhance the negative impact of CdB concentration.

Numerous studies have shown that the consumption of calcium could decrease the absorption of lead through the digestive tract because of competition at the level of the gastrointestinal receptors [43, 44]. Furthermore, animal studies indicate a possible dose-dependent relationship between calcium supplement levels and the reduction of blood lead level [45, 46]. Some human studies demonstrated that high milk intake significantly reduces lead toxicity of the sensory nervous system in lead-exposed workers [47]. Besides, Liu and Zhang [48] indicated that low serum Ca^2+^ levels are related with high blood lead levels in male lead-exposed workers. Some studies showed a significant inverse association between dietary Ca intake and PbB levels, whereas there was no significant association with concomitant CdB levels in nonsmoking females [49] and in lead battery factory workers (PbB: 41.4 *μ*g/dl; [50]). In the present study, the levels of lead in the blood were not different between the L-Ca group and the H-Ca group; however, there was a negative correlation between PbB and Ca concentration. Besides, the current study establishes that the level of ZPP was higher in the L-Ca group than in the H-Ca group and calcium level negatively correlates with ZPP level. Lead inhibits ferrochelatase activity and therefore prevents incorporation of iron into hemoglobin. This reaction also leads to the binding of zinc, producing ZPP [51]. The ZPP concentration has been commonly used in clinical diagnosis of chronic occupational lead exposure [52–54]. Mazumdar et al. [55] showed that ZPP levels are negatively correlated with serum calcium concentration in jewelry makers (PbB: 69 *μ*g/dl). Our observation suggests that low calcium levels may enhance the negative impact of lead on heme biosynthesis. On the other hand, the impact of Ca concentration on blood cells seems to be limited; we simultaneously did not observe any differences between the examined groups in terms of the values of RBC count, HTC, and HGB level. Similarly, there was no difference between the values of the WBC counts and PLT level in the L-Ca group compared to the H-Ca group.

Many findings on the oxidative damage to various biological macromolecules caused by exposure to Pb or Cd suggest that even though these metals are nonredox, they can cause disturbances in oxidative status that can significantly contribute to many adverse effects of these two toxic metals. The most important biochemical change associated with cadmium exposure is upregulation of metallothionein, a cysteine-rich, metal-binding protein that sequesters Cd and also possesses considerable free radical scavenging ability [13].

Pb^2+^ has been shown to be associated with increased generation of ROS. Besides, lead is able to dysregulate the antioxidant defenses, including the antioxidant enzymes and the nonenzymatic antioxidants [56]. Lipid peroxidation in red blood cell membranes as a consequence of Pb-induced oxidative stress leads to hemolysis and contributes to Pb-induced anemia [57]. The oxidative stress levels can disrupt normal physiological Ca^2+^ signaling pathways. ROS induce Ca^2+^ influx into the cytoplasm from the extracellular environment and rising Ca^2+^ concentration in the cytoplasm, mitochondria, and nuclei. High cellular calcium level accelerates and dysregulates phosphorylation/dephosphorylation of proteins and disrupts normal metabolism [58]. An *in vitro* study has indicated that lead toxicity leads to the increase of intracellular Ca^2+^ and ROS formation in PC12 cells [59]. Moreover, animal studies demonstrated that calcium sublimation reduces MDA level in lead-exposed rats [60] and mice [46]. Chen et al. [59] observed that lipid peroxidation levels in blood were strongly correlated (dose-dependent relationship) with blood lead concentration in exposed workers. The current study establishes that the L-Ca group had higher serum MDA level compared to the H-Ca group. Besides, we reported a negative correlation between calcium concentration and MDA level in examined groups. These data support the hypothesis that Ca may play an important role in the prevention of related oxidative damage of blood erythrocytes. On the other hand, our results did not show any relationships between calcium level and the activity of antioxidant enzymes. We can only speculate that this lack of relationship is caused by a small difference in Ca concentration in the study groups.

Ceruloplasmin (CER) is a member of the multicopper oxidase family of enzymes that includes ascorbate oxidase and the laccases. It is an acute-phase protein that can convert ferrous iron to its less reactive ferric form facilitating binding to ferritin. Bento et al. [61] indicated that CER has a Ca^2+^-binding site that appears to be an integral component of the structure and may play a role in the interactions of the protein with red blood cells. Some experimental studies demonstrated that lead exposure was able to induce significant alterations in serum ceruloplasmin oxidase activity; however, Ca supplementation did not reverse the Pb-induced alterations in rats [60]. There is no evidence that shows a relationship between Cd exposure, CRP level, and Ca concentration. Gümüşlü et al. [62] showed that serum CER level was unaltered after Cd exposure and antioxidant treatment in rats. The results of the present study revealed that CER level was higher in the L-Ca group compared to the H-Ca group. Perhaps, calcium can interact with acute-phase proteins and reduce their level in serum.

## 5. Conclusions

The presented results indicate that Ca level modulates the serum concentration of Cd and has an impact on lead-induced impairment of heme synthesis. The higher Ca level may lead to a decrease in the concentration of lipid peroxidation products. Moreover, the serum Ca level seems to be able to modify the level of acute-phase proteins. The obtained results suggest that a higher Ca level may be useful in reducing the Cd level in occupationally exposed workers.

## Figures and Tables

**Table 1:** Blood morphology parameters in the low-calcium-level group (L-Ca group) and the high-calcium-level group (H-Ca group).

	L-Ca group		H-Ca group		Relative change %	*p*
	Mean	±SD	Mean	±SD		
WBC (G/l)	7.12	1.86	7.37	2.11	4%	0.304
RBC (T/l)	15.2	0.86	15.2	0.89	0%	0.985
HGB (g/dl)	43.0	2.28	43.1	2.52	0%	0.665
HTC (%)	234	50.5	228	57.20	-2%	0.419
PLT (G/l)	15.2	0.86	15.2	0.89	0%	0.985

WBC: white blood cells. RBC: red blood cells. HGB: hemoglobin. HTC: hematocrit. PLT: platelets. Data are expressed asmean ± standard error.

**Table 2:** The concentrations of malondialdehyde (MDA), lipofuscin (LPS), glutathione reductase (GR), glutathione S-transferase (GST), glutathione peroxidase (GPX), catalase (CAT), and superoxide dismutase (SOD) in erythrocytes in the low-calcium-level group (L-Ca group) and the high-calcium-level group (H-Ca group).

	L-Ca group		H-Ca group		Relative change %	*p*
	Mean	±SD	Mean	±SD		
MDA (nmol/gHb)	219	62.63	232	77.91	6%	0.140
LPS (RU/gHb)	654	217	638	280	-2%	0.603
GR (IU/gHb)	7.00	1.55	6.96	1.44	-1%	0.826
GST (mIU/gHb)	184	72.0	185	76.60	1%	0.906
GPX (IU/gHb)	52.2	13.6	50.5	15.72	-3%	0.357
CAT (kIU/gHb)	458	63.5	462	65.16	1%	0.653
SOD (NU/mgHb)	192	33.5	198	35.45	3%	0.141

Data are expressed asmean ± standard error.

**Table 3:** Correlations between calcium level and selected parameters.

Parameters	*R*	*p*
PbB (*μ*g/dl)	**-0.13**	**0.034**
ZPP (*μ*g/gHb)	**-0.16**	**0.008**
CdB (*μ*g/l)	**-0.24**	**0.001**
MDA (*μ*mol/l)	**-0.14**	**0.025**
CER (mmol/l)	-0.10	0.123

PbB: blood lead level. ZPP: zinc protoporphyrin. CdB: blood cadmium level. MDA: malondialdehyde. CER: ceruloplasmin.*R*values: Spearman's rank correlation.

## Data Availability

The data used to support the findings of this study are available from the corresponding author upon request.
